# Ameliorating effect of *Mucuna pruriens* seed extract on sodium arsenite-induced testicular toxicity and hepato-renal histopathology in rats

**DOI:** 10.14202/vetworld.2023.82-93

**Published:** 2023-01-11

**Authors:** Preethi Lavina Concessao, Kurady Laxminarayana Bairy, Archana Parampalli Raghavendra

**Affiliations:** 1 Department of Physiology, Department of Basic Medical Sciences, Manipal Academy of Higher Education, Manipal, Karnataka, India; 2 Department of Pharmacology, RAK College of Medical Sciences, RAK Medical and Health Sciences University, Ras Al Khaimah, United Arab Emirates

**Keywords:** arsenic, DNA damage, hepato-renal, *Mucuna pruriens*, testis damage

## Abstract

**Background and Aim:**

A significant cause of arsenic poisoning is polluted groundwater. Arsenic poisoning results in the suppression of spermatogenesis and the liver and kidneys are vulnerable to the toxic effects as well. *Mucuna pruriens* has been identified to have fertility-enhancing and anti-lipid peroxidation properties. Based on these properties of *M. pruriens*, this study aimed to investigate the efficacy of *M. pruriens* seed extract in reducing sodium arsenite-induced testicular impairment and hepato-renal histopathology in rats.

**Materials and Methods:**

The study was divided into two groups; short-term (45 days) and long-term (90 days) treatment groups and each group was divided into nine subgroups. Subgroups 1 and 2 served as normal and N-acetyl cysteine (NAC) controls, respectively. Subgroups 3–9 received sodium arsenite in the drinking water (50 mg/L). Subgroup-4 received NAC (210 mg/kg body weight [BW]) orally once daily. Subgroups 5–7 received aqueous seed extract of *M. pruriens* (350, 530, and 700 mg/kg BW, respectively) orally once daily. Subgroups 8 and 9 received a combination of NAC and aqueous seed extract (350 and 530 mg/kg BW, respectively) orally once daily. Following the treatment, animals were sacrificed and sperm parameters and DNA damage were evaluated. Testis, liver, and kidneys were analyzed for histopathology.

**Results:**

Sodium arsenite-induced a significant reduction in sperm parameters and increase in the abnormal architecture of spermatozoa. Histology revealed tissue necrosis. The *M. pruriens* seed extract ameliorated the damaging effects of sodium arsenite with respect to tissue architecture and sperm parameters when coadministered.

**Conclusion:**

*Mucuna prurien*s has beneficial effects against the deleterious effects of sodium arsenite on various tissues. Thus, *M. pruriens* (530 and 700 mg/kg BW) supplementation would reduce the adverse changes observed with sodium arsenite exposure.

## Introduction

Arsenic is an all-pervading metalloid found in the earth’s crust and exists in elemental, organic, and inorganic forms. Contaminated groundwater is the major cause of arsenic poisoning [1]. Arsenic predominantly disturbs the sulfhydryl groups in cells [2], affecting cell respiration, enzymes, and mitosis [3, 4]. The human body is exposed to arsenic through different avenues, such as ingestion, air inhalation, and absorption through the skin [5]. Arsenic affects male sex organs and can lead to reproductive issues. It may disrupt gonadal function by decreasing testosterone synthesis [6], and induces apoptosis and necrosis [7, 8]. Studies have demonstrated a reduction in the weight of the reproductive organs and steroidogenesis with elevated levels of arsenic in the testis, indicating reproductive toxicity [9–11]. Toxicity due to arsenic was also shown to result in injury to the liver cells, fatty degradation, and progressive fibrosis [12, 13]. Several metal chelating agents have been previously tested and reported, including N-acetyl cysteine (NAC). It is a thiol and a precursor of reduced glutathione that serves as a free radical scavenger since it interacts with reactive oxygen species.

Several reports have shown that herbs and plant products could be used to mitigate arsenic toxicity. *Mucuna pruriens* is a leguminous plant that possesses antidiabetic and aphrodisiac effects [14, 15]. Seeds of *M. pruriens* have been shown to improve semen quality, testosterone, and luteinizing hormone levels [16], re-activate the antioxidant defense systems, and reduce stress [17, 18]. The existing literature established that excessive arsenic exposure causes oxidative cellular damage, which leads to organ damage. *Mucuna prurien*s serves as a source of natural antioxidants, reducing the damage caused by oxidative stress. However, there are no available reports on the use of *M. pruriens* seed extract to possibly reduce or prevent systemic toxicity induced by arsenic.

This study aimed to determine the effects of *M. pruriens* against sodium arsenite-induced sperm abnormalities and histopathological changes in the liver, kidney, and testes of rats.

## Materials and Methods

### Ethical approval

All procedures used in this study were approved by the Animal Ethics Committee of Manipal Academy of Higher Education (IAEC/KMC/52/2015).

### Study period and location

This study was conducted from December 2018 to August 2019 at Animal House of Manipal Academy of Higher Education and at the Laboratory of Melaka Manipal Medical College, Manipal.

### Chemicals

- Sodium arsenite A.R (98.5%) was obtained from Nice Chemicals (P) Ltd., Cochin, India. NAC (Samarth Life Sciences Private Limited, India) was procured from a medical store at Udupi.
- Hematoxylin stain, Eosin Y stain and Cresyl violet powder were obtained from Karnataka laboratories, Mangalore, India.

### Plant extract preparation

Identification of *M. pruriens* seeds was conducted by the Faculty of Pharmacognosy (Specimen No: SDM/954/17112301). Seeds of *M. pruriens* were collected locally and cleaned. A total of 100 g of powdered seeds was soaked in 1000 mL distilled water at 4°C for 8 d. The suspension was centrifuged at 10000× g for 25 min and the supernatant was removed and used as the extract [19].

### Extracts quality assessment

The *M. pruriens* seed extract was qualitatively checked for the identification of carbohydrates, saponins, phenolic compounds, tannins, flavonoids, proteins, amino acids, gums, and mucilage. The physiochemical parameters were assessed for the standardization of each batch of prepared extract. The yield from the extract was 15.8 g.

### Experimental animals

One hundred and eight Sprague Dawley male rats (9–12 weeks old) were selected for the study and locally bred at the central animal house of Manipal Academy of Higher Education, Manipal. They were individually housed in polypropylene cages containing sterile paddy husk (locally procured) as bedding throughout the study and maintained under standard conditions with the temperature at 22°C–24°C, a 12 h/12 h light/dark cycle, and 40%–60% relative air humidity. The animals were acclimatized to the laboratory conditions for one week before the start of the experiment. Breeding and maintenance of animals were performed in accordance with the guidelines of the Committee for control and supervision of experiments on animals and Animal Welfare Division, Government of India for the use of laboratory animals. Three animals were housed in each cage to prevent overcrowding. Standard laboratory feed and water were available *ad libitum* to the animals.

### Experimental design

The study was divided into two groups: short-term (45 days) and long-term (90 days) treatment groups.

#### Short-term group

Subgroup-1 (normal control) received normal drinking water as a vehicle for 45 days.

Subgroup-2 (standard drug NAC) received NAC at a dose of 210 mg/kg body weight (BW) once per day for 45 days.

Subgroup-3 (sodium arsenite - toxic control) received sodium arsenite in the drinking water (50 mg/L) for 45 days [20].

Subgroup-4 (sodium arsenite + standard drug NAC) received sodium arsenite in drinking water (50 mg/L) + NAC at a dose of 210 mg/kg BW [21] once per day for 45 days.

Subgroup-5 (sodium arsenite + *M. pruriens)* received sodium arsenite in drinking water (50 mg/L) + *M. pruriens* at a dose of 350 mg/kg BW [22] orally once per day for 45 days.

Subgroup-6 (sodium arsenite + *M. pruriens*) received sodium arsenite in drinking water (50 mg/L) + *M. pruriens* at a dose of 530 mg/kg BW orally once per day for 45 days.

Sub group-7 (sodium arsenite + *M. pruriens*) received sodium arsenite in drinking water (50 mg/L) + *M. pruriens* at a dose of 700 mg/kg BW orally once per day for 45 days.

Subgroup-8 (sodium arsenite + NAC + *M. pruriens*) received sodium arsenite in drinking water (50 mg/L) + NAC at a dose of 210 mg/kg BW + *M. pruriens* at a dose of 350 mg/kg BW orally once per day for 45 days.

Subgroup-9 (sodium arsenite + NAC + *M. pruriens*) received sodium arsenite in drinking water (50 mg/L) + NAC at a dose of 210 mg/kg BW + *M. pruriens* at a dose of 530 mg/kg BW orally once per day for 45 days.

#### Long-term group

Subgroup-1 (normal control) received drinking water as a vehicle for 90 days.

Subgroup-2 (standard drug NAC) received NAC at a dose of 210 mg/kg BW once per day for 90 days.

Subgroup-3 (sodium arsenite − toxic control) received sodium arsenite in drinking water (50 mg/L) for 90 days.

Subgroup-4 (sodium arsenite + standard drug NAC) received sodium arsenite in drinking water (50 mg/L) + NAC at a dose of 210 mg/kg BW once per day for 90 days.

Subgroup-5 (sodium arsenite + *M. pruriens*) received sodium arsenite in drinking water (50 mg/L) + *M. pruriens* at a dose of 350 mg/kg BW orally once per day for 90 days.

Subgroup-6 (sodium arsenite + *M. pruriens*) received sodium arsenite in drinking water (50 mg/L) + *M. pruriens* at a dose of 530 mg/kg BW orally once per day for 90 days.

Subgroup-7 (sodium arsenite + *M. pruriens*) received sodium arsenite in drinking water (50 mg/L) + *M. pruriens* at a dose of 700 mg/kg BW orally once per day for 90 days.

Subgroup-8 (sodium arsenite + NAC + *M. pruriens*) received sodium arsenite in drinking water (50 mg/L) + NAC at a dose of 210 mg/kg BW + *M. pruriens* at a dose of 350 mg/kg BW orally once per day for 90 days.

Subgroup-9 (sodium arsenite + NAC + *M. pruriens*) received sodium arsenite in drinking water (50 mg/L) + NAC at a dose of 210 mg/kg BW + *M. pruriens* at a dose of 530 mg/kg BW orally once per day for 90 days.

Following the treatment, the animals were sacrificed and the sperm parameters and DNA damage were assessed. Testis, liver, and kidneys were analyzed for histopathology.

### Investigated parameters

Epididymal sperm count, sperm motility, and sperm morphology tests were performed as described by Vega *et al*. [23].

The sperm chromatin dispersion test was performed as described by Kumari *et al*. [24].

Histopathological evaluation of the kidneys, liver, and testis was performed.

All animals in the experimental groups were euthanized after treatment. The liver, testis, and kidneys were dissected and fixed with formalin (10%). The tissues were processed for paraffin sectioning and stained with Hematoxylin and Eosin. The presence of vacuoles, gaps, and aberrant cells was examined in stained tissues.

### Quantitative analysis

#### Seminiferous tubular diameter

The diameters of 10 transversely cut round seminiferous tubules were randomly selected per animal and measured using an ocular micrometer calibrated with a stage micrometer. In each tubule, two measurements were taken, one perpendicular to the other and their average was determined.

#### Seminiferous epithelial height

In a similar manner to the seminiferous tubular diameter measurement, the epithelial height was measured in 10 tubules from the basement membrane to the surface of the epithelium in two distinct areas, and the average was calculated.

### Statistical analysis

Data were analyzed using a one-way analysis of variance followed by a *post hoc* Tukey test with Prism Version 5.0 (trial version). Results were expressed as mean ± standard deviation. p ≤ 0.05 was considered as significant.

## Results

### Phytochemical screening of the *M. pruriens* aqueous seed extract

The yield from the aqueous seed extract of *M. pruriens* was 15.8 g. The seed extract was subjected to primary phytochemical investigation and the presence of alkaloids, carbohydrates, saponins, tannins, flavonoids, and phenols was detected (Table-1).

**Table-1:** Phytoconstituents present in aqueous seed extract of *Mucuna pruriens.*

Test	Inference
Alkaloid	+++
Carbohydrate	+++
Flavonoids	+
Saponins	++
Tannin	+++
Terpenoid	-
Protein	-
Phenol	++
Steroid	+

### Sperm count

There was a significant reduction (p < 0.001) in the sperm count after treatment with sodium arsenite compared to the normal control group. Co-administration of *M. pruriens* (350, 530, and 700 mg/kg BW) along with sodium arsenite significantly increased (p < 0.01) the sperm count in comparison to the sodium arsenite-only treated group following both treatment durations. The groups treated with a combination of sodium arsenite + NAC along with *M. pruriens* (350 [p < 0.05] and 530 mg/kg BW [p < 0.01]) exhibited an increase in sperm count when compared to the sodium arsenite-only treatment group (Tables-2 and 3).

**Table-2:** Effect of *Mucuna pruriens* treatment (45 days) on sperm count, sperm motility, sperm abnormality, seminiferous tubular diameter, and seminiferous epithelial height of arsenic exposed animals.

Groups	Sperm count (millions/mL)	Sperm motility (%)	Sperm abnormality (%)	Seminiferous tubular diameter (microns)	Seminiferous epithelial height (microns)
Control	61.18 ± 10.05	24.74 ± 2.184	12.36 ± 1.62	180.41 ± 4.72	47.5 ± 2.14
NAC	58.87 ± 4.27	23.91 ± 2.05	12.41 ± 1.75	181.25 ± 3.79	50 ± 2.75
As control	32.38 ± 2.47^$a^	15.22 ± 0.88^$a^	36.5 ± 1.94^$a^	227.6 ± 3.07^$a^	36.25 ± 0.27^$a^
As+NAC	44.23 ± 2.29^#b^	19.64 ± 1.44^$b^	24.6 ± 2.4^$b^	206.15 ± 6.44	42.5 ± 1.53^$b^
As+MP (350)	40.79 ± 1.97*^b^	18.92 ± 1.29*^b^	31.2 ± 2.84*^b^	215.14 ± 5.61	37.50 ± 2.70
As+MP (530)	42.85 ± 1.77^#b^	19.39 ± 2.07^#b^	26.05 ± 2.71^$b^	211.97 ± 7.42	38.75 ± 1.30
As+MP (700)	43.79 ± 2.57^#b^	19.61 ± 0.71^#b^	25.38 ± 3.7^$b^	208.57 ± 3.54	42.5 ± 2.9^$b^
As+NAC+MP (350)	41.67 ± 1.50*^b^	19.01 ± 1.16^#b^	24.06 ± 1.16^$b^	208.25 ± 3.22	41.25 ± 2.76^$b^
As+NAC+MP (530)	43.47 ± 1.98^#b^	19.02 ± 1.97^#b^	31.52 ± 1.52*^b^	207.00 ± 5.4	40 ± 1.3

Values are mean ± SD, (n=6) in each group.

* p < 0.05,

# p < 0.01,

$ p < 0.001.

a Compared to normal control,

b Compared to As control. NAC=N-acetyl cysteine, As=Arsenic, MP (350)=*Mucuna pruriens* aqueous extract 350 mg/kg body weight, MP (530)=*Mucuna pruriens* aqueous extract 530mg/kg body weight, MP (700)=*Mucuna pruriens* aqueous extract 700mg/kg body weight

**Table-3:** Effect of *Mucuna pruriens* treatment (90 days) on sperm count, sperm motility, sperm morphology, seminiferous tubular diameter, and seminiferous epithelial height of arsenic exposed animals

Groups	Sperm count (millions/mL)	Sperm motility (%)	Sperm abnormality (%)	Seminiferous tubular diameter (microns)	Seminiferous epithelial height (microns)
Control	61.88 ± 8.51	24.24 ± 1.19	12.44 ± 1.81	182.76 ± 3.79	50 ± 2.14
NAC	59.53 ± 2.49	23.51 ± 1.70	10.11 ± 0.93	181.25 ± 6.97	51.25 ± 2.30
As control	30.72 ± 1.67^$a^	13.54 ± 0.81^$a^	39.23 ± 2.77^$a^	240.08 ± 2.43^$a^	26.25 ± 1.24^$a^
As+NAC	42.47 ± 2.314^$b^	19.46 ± 1.31^$b^	18.7 ± 3.4^$b^	215.84 ± 9.41^$b^	41.25 ± 0.70^$b^
As+MP (350)	40.33 ± 2.65^#b^	16.90 ± 1.87^#b^	26.81 ± 1.23^$b^	222.94 ± 6.22^#b^	35 ± 2.70^$b^
As+MP (530)	42.49 ± 2.07^$b^	17.11 ± 1.45^#b^	24.1 ± 3.49^$b^	209.76 ± 7.78^$b^	40 ± 1.63^$b^
As+MP (700)	42.27 ± 1.99^$b^	18.55 ± 1.29^$b^	20.32 ± 2.08^$b^	211.09 ± 7.07^$b^	41.25 ± 1.53^$b^
As+NAC+MP (350)	44.06 ± 5.90^$b^	17.20 ± 1.32^#b^	24.16 ± 2.46^$b^	214.00 ± 2.07^$b^	41.25 ± 1.70^$b^
As+NAC+MP (530)	43.9 ± 6.49^$b^	17.92 ± 1.51^$b^	24.82 ± 3.25^$b^	214.15 ± 3.71^$b^	40 ± 0.1^$b^

Values are mean ± SD, (n=6) in each group.

# p < 0.01,

$ p < 0.001.

a Compared to normal control,

b Compared to As control. NAC=N-acetyl cysteine, As=Arsenic, MP (350)=*Mucuna pruriens* aqueous extract 350 mg/kg body weight, MP (530)=*Mucuna pruriens* aqueous extract 530 mg/kg body weight, MP (700)=*Mucuna pruriens* aqueous extract 700 mg/kg body weight

### Sperm motility

The number of motile sperm significantly decreased (p < 0.001) in the sodium arsenite-only treated group compared to the normal control. Co-administration of *M. pruriens* (350, 530, and 700 mg/kg BW) along with sodium arsenite significantly increased (p < 0.01) the number of motile sperm in comparison to the sodium arsenite-only treated group. Sperm motility significantly increased in the animals treated with a combination of sodium arsenite + NAC along with *M. pruriens* (350 and 530 mg/kg BW [both p < 0.01]) in comparison to the sodium arsenite-only treated group (Tables-2 and 3).

### Sperm morphology

Exposure to sodium arsenite significantly increased (p < 0.001) the number of abnormal sperm in comparison to the normal control. Co-administration of *M. pruriens* (350 [p < 0.05], 530 [p < 0.001], and 700 mg/kg BW [p < 0.001]) along with sodium arsenite significantly decreased the number of abnormal sperms in comparison to the sodium arsenite only treatment group. Animals treated with sodium arsenite + NAC + *M. pruriens* (350 [p < 0.05] and 530 [p < 0.001] mg/kg BW) showed a significant decrease in the number of abnormal sperms when compared to sodium arsenite + NAC treatment group (Tables-2 and 3).

### Seminiferous tubular diameter

Animals treated with sodium arsenite showed a significant increase (p < 0.001) in the seminiferous tubular diameter, indicating a distortion of the tubule when compared to the normal controls. In the long-term treatment group, co-administration of sodium arsenite + *M. pruriens* (350, 530, and 700 mg/kg BW [p < 0.001]) and co-administration of sodium arsenite + NAC+ *M. pruriens* (350 and 530 mg/kg BW [p < 0.001]) exhibited a significant decrease in the seminiferous tubular diameter when compared to sodium arsenite only treated group (Tables-2 and 3).

### Seminiferous epithelial height

Sodium arsenite administration caused a significant reduction (p < 0.001) in the seminiferous epithelial height when compared to the normal control group. In the short-term treatment group, animals treated with sodium arsenite + *M. pruriens* 700 mg/kg BW had a significantly increased (p < 0.001) seminiferous epithelial height when compared to the sodium arsenite-only treatment group. Animals treated with sodium arsenite + NAC + *M. pruriens* (350 mg/kg BW) showed a significant increase (p < 0.001) in seminiferous epithelial height when compared to the sodium arsenite-only treated group. In the long-term treatment group, co-administration of sodium arsenite + *M. pruriens* (350, 530, and 700 mg/kg BW [p < 0.001]) and co-administration of sodium arsenite + NAC + *M. pruriens* (350 and 530 mg/kg BW [p < 0.001]) exhibited a significant increase in the seminiferous epithelial height when compared to sodium arsenite only treated group (Tables-2 and 3).

### Sperm DNA damage

In the group treated with sodium arsenite alone, a significant increase (p < 0.001) in the percentage of spermatozoa carrying DNA damage when compared to normal control was observed. Animals treated with a combination of *M. pruriens* (530 and 700 mg/kg BW [p < 0.001]) along with sodium arsenite showed a reduction in the spermatozoa containing damaged DNA in comparison to the sodium arsenite-only group. The spermatozoa from the groups treated with a combination of NAC and *M. pruriens* (350 and 530 mg/kg BW [p < 0.001]), along with sodium arsenite, also exhibited a significant reduction in the percentage of DNA damage (Table-4).

**Table-4:** Effect of *Mucuna pruriens* treatment (45 and 90 days) on sperm DNA damage of arsenic exposed animals.

Groups	Sperm DNA damage (%)	

	45 days treatment	90 days treatment
Control	6.67 ± 0.88	9 ± 1.57
As control	24.66 ± 0.88^$a^	30.33 ± 1.21^$a^
As+NAC	10 ± 2.08^$b^	13 ± 2.08^$b^
As+MP (530)	16.6 ± 1.45^$b^	17.33 ± 2.96^$b^
As+MP (700)	12 ± 2.31^$b^	15 ± 1.52^$b^
As+NAC+MP (350)	13.33 ± 2.34^$b^	18 ± 5.81^$b^
As+NAC+MP (530)	13 ± 2.85^$b^	20 ± 1.85^#b^

Values are mean ± SD, (n=4) in each group.

# p < 0.01,

$ p < 0.001,

a Compared to normal control,

b Compared to As control. NAC=N-acetyl cysteine, As=Arsenic, MP (350)=*Mucuna pruriens* aqueous extract 350 mg/kg body weight, MP (530)=*Mucuna pruriens* aqueous extract 530 mg/kg body weight, MP (700)=*Mucuna pruriens* aqueous extract 700 mg/kg body weight

### Histopathology of the testis

**Figure-1:**
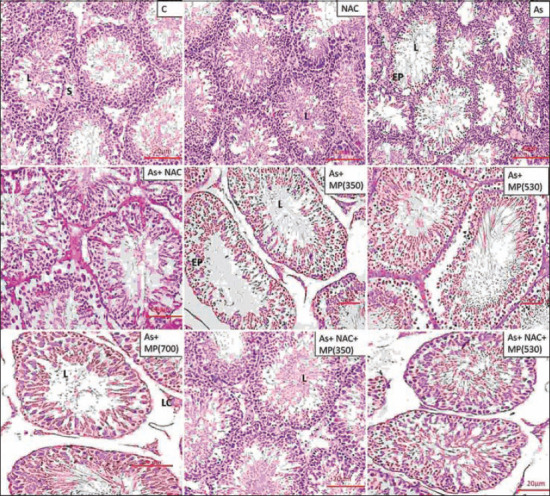
Representative photographs of H and E stained sections of testis following 45 days of treatment, viewed under 100×. Scale bar=20 μm. NAC=N-acetyl cysteine, As=Arsenic, MP (350)=*M. pruriens* aqueous seed extract 350 mg/kg BW, MP (530)=*M. pruriens* aqueous seed extract 530 mg/kg body weight, MP (700)=*M. pruriens* aqueous seed extract 700 mg/kg body weight. Control group and NAC group showed normal organization of seminiferous tubule. Arsenic treated group showed reduced seminiferous tubule epithelial height and increased tubular lumen. The tubular basement membrane was irregular or interrupted. There was less number of spermatozoa in the lumen. As + MP (350) and As + MP (530) treated group exhibited cellular damage (arrow) and Sertoli cell damage (arrow). Higher doses of *Mucuna pruriens* and the combination treatment showed improved cell count, decreased structural damage. L=Lumen, E=Epithelium, S=Stroma.

**Figure-2:**
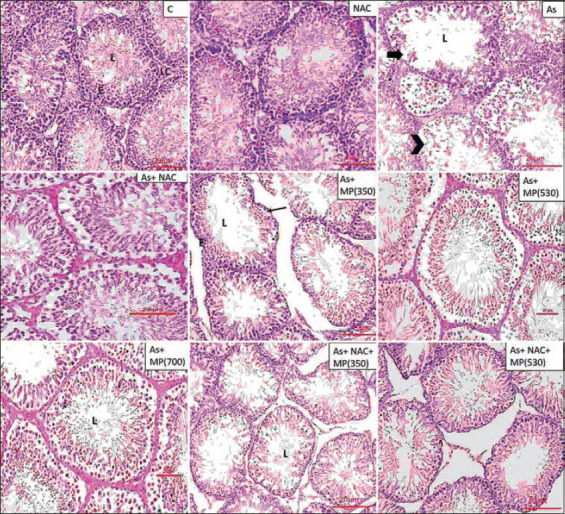
Representative photographs of H and E stained sections of testis following 90 days of treatment, viewed under 100×. Scale bar=20 μm. NAC=N-acetyl cysteine, As=Arsenic, MP (350)=*Mucuna pruriens* aqueous seed extract 350 mg/kg body weight, MP (530)=*Mucuna pruriens* aqueous seed extract 530 mg/kg body weight, MP (700)=*Mucuna pruriens* aqueous seed extract 700 mg/kg body weight. Control group and NAC group showed normal organization of seminiferous tubule. Arsenic treated group showed reduced seminiferous tubule epithelial height and increased tubular lumen. The tubular basement membrane was irregular or interrupted (arrow). Less spermatozoa in the lumen. Provoked alterations in the seminiferous tubules were evident. Sloughing of germ cells was seen (arrow head). As+ MP (350) treated group showed intact basement membrane (arrow), but reduction in the cell count in the lumen. The higher doses of *Mucuna pruriens* (530 mg/kg body weight and 700 mg/kg body weight) and the combination treatments showed minimal damage and improved cell count. L=Lumen, E=Epithelium, LC=Leydig cell.

The testicular architecture of the control and NAC groups showed normal organization of the seminiferous tubule. Sodium arsenite administration (45 and 90 days) showed a significant reduction in seminiferous tubule diameter, seminiferous tubule epithelial height, and increased tubular lumen. Sloughed germ cells with no maturation stages were observed and the tubular basement membrane was irregular or interrupted. The cell lining of Sertoli cells was damaged and in general, there were fewer spermatozoa observed in the central core of the tubule. Co-administration of *M. pruriens* (350 and 530 mg/kg BW) along with sodium arsenite minimized the toxic effect of arsenic on the testis. The combination of NAC + *M. pruriens* (350 and 530 mg/kg BW) along with sodium arsenite showed improved structural recovery with minimal damage and intact cellular structures with an improved cell count (Figures-1 and -2).

### Histopathology of the liver

**Figure-3:**
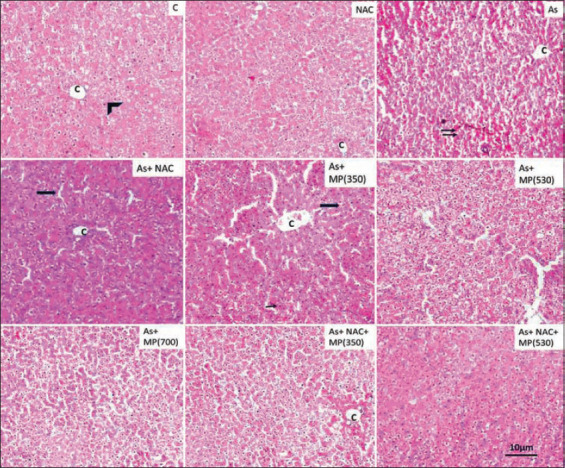
Representative photographs of H and E stained sections of liver following 45 days of treatment, viewed under 100×. Scale bar=10 μm. NAC=N-acetyl cysteine, As=Arsenic, MP (350)=*Mucuna pruriens* aqueous seed extract 350 mg/kg body weight, MP (530)=*Mucuna pruriens* aqueous seed extract 530 mg/kg body weight, MP (700)=*Mucuna pruriens* aqueous seed extract 700 mg/kg body weight. Normal histoarchitecture with normal hepatocytes (arrow head), sinusoids, central vein (C) and Kupffer cells were observed in control and the NAC treated groups. Arsenic treated group showed damage to the cell lining of sinusoids, increase in sinusoidal spaces and dilated central vein. Wall of the central vein was damaged. Cellular necrosis of hepatocytes was seen (double arrow). As + MP (350) treated group showed inflammatory changes in the hepatocytes along with increase in the area of sinusoids (arrow) and dilated central vein.

**Figure-4:**
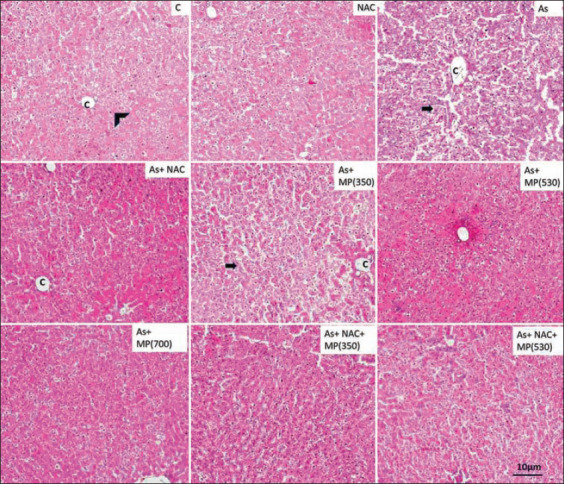
Representative photographs of H and E stained sections of liver following 90 days of treatment, viewed under 100×. Scale bar=10 μm. NAC=N-acetyl cysteine, As=Arsenic, MP (350)=*Mucuna pruriens* aqueous seed extract 350 mg/kg body weight, MP (530)=*Mucuna pruriens* aqueous seed extract 530 mg/kg body weight, MP (700)=*Mucuna pruriens* aqueous seed extract 700 mg/kg body weight. Normal histoarchitecture with normal hepatocytes (arrow head), central vein (C), sinusoids, and Kupffer cells were observed in control and the NAC treated groups. Arsenic treated group showed damage to the cell lining of sinusoids, increase in sinusoidal spaces (arrow). Cellular necrosis of hepatocytes was seen (double arrow). As + MP (350) treated group showed macrophage activity. Increased sinusoidal spaces were evident (arrow).

The liver of the normal group showed hepatocytes that were arranged in strands radiating from the central vein and sinusoids and Kupffer cells were observed. Exposure to sodium arsenite for 45 days resulted in damage to the cell lining of sinusoids and an increase in sinusoidal spaces. The wall of the central vein was damaged and appeared to be dilated. Long-term exposure resulted in damage that was extended to other areas of the liver. Disorganization of hepatic lobules and cellular necrosis of hepatocytes was evident. In the animals exposed to sodium arsenite + NAC, there were major changes in the sinusoidal regions. The higher doses of *M. pruriens* (530 and 700 mg/kg BW) showed improved cellular structure. Clear sinusoidal regions were evident. Inflammatory changes were reduced. The combination of *M. pruriens* and + NAC showed reduced inflammatory and degenerative changes due to sodium arsenite (Figures-3 and -4).

### Histopathology of the kidney

**Figure-5:**
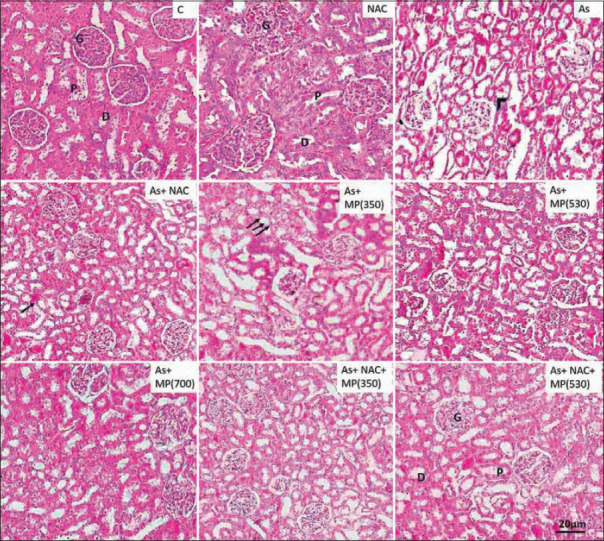
Representative photographs of H and E stained sections of kidney following 45 days of treatment, viewed under 100×. Scale bar=20 μm. NAC=N-acetyl cysteine, As=Arsenic, MP (350)=*Mucuna pruriens* aqueous seed extract 350 mg/kg body weight, MP (530)=*Mucuna pruriens* *Mucuna pruriens* aqueous seed extract 530 mg/kg body weight, MP (700)=*Mucuna pruriens* aqueous seed extract 700 mg/kg body weight. The kidney tissue of control and NAC treated rats showed normal renal corpuscle (G), proximal convoluted tubules (P), distal convoluted tubules (D). In the arsenic treated rats, renal cortex swelling and congestion in the renal corpuscle (arrow), showed damage to the bowman capsule, basement membrane, increased urinary space, damage to the intercellular structures (arrow head). As + NAC treated group showed inflammatory changes (arrow). As + MP (350) treated group showed infiltration of inflammatory cells (double arrow) and macrophage activity (arrow).

**Figure-6:**
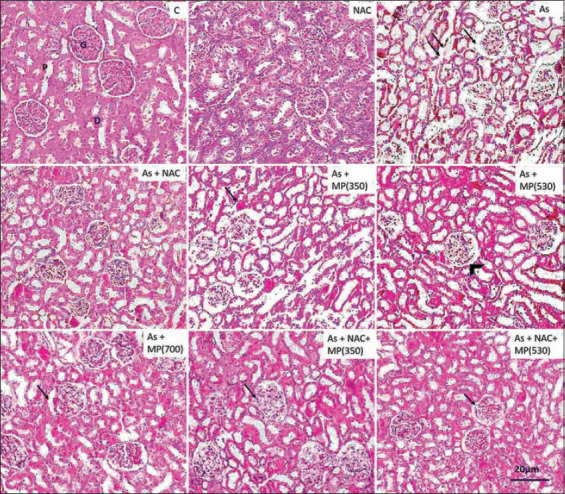
Representative photographs of H and E stained sections of kidney following 90 days of treatment, viewed under 100×. Scale bar=20 μm. NAC=N-acetyl cysteine, As=Arsenic, MP (350)=*Mucuna pruriens* aqueous seed extract 350 mg/kg body weight, MP (530)=*Mucuna pruriens* aqueous seed extract 530 mg/kg body weight, MP (700)=*Mucuna pruriens* aqueous seed extract 700 mg/kg body weight. The kidney tissue of control and NAC treated rats showed normal renal corpuscle (G), proximal convoluted tubules (P), distal convoluted tubules (D). Arsenic treated rats showed damage to the bowman capsule (arrow), basement membrane increased urinary space, damage to the intercellular structures (double arrow). As + MP (350) treated group showed damage to the cellular lining of the duct system. As + MP (530) group showed reduction in the urinary space (arrow head).

Sections from kidney tissue exposed to sodium arsenite showed damage to the bowman capsule leading to increased urinary space. There was clear damage to the basement membrane and intercellular structures. Co-administration of *M. pruriens* (350 mg/kg BW) with sodium arsenite reduced the toxic effects of arsenic. There was damage to the cellular lining of the duct system. Higher doses of *M. pruriens* (530 and 700 mg/kg BW) showed minimal damage and an improved histological structure in comparison to the lower dose. The kidney tubules exhibited acidophilic cytoplasm. The combination of *M. pruriens* (350 and 530 mg/kg BW) + NAC showed results that were similar to sodium arsenite *+ M. pruriens* (700 mg/kg BW) (Figures-5 and -6).

## Discussion

In this study, a significant reduction was observed in the sperm count and motility and an increase in aberrant sperm in the animals exposed to sodium arsenite in both treatment periods when compared to the normal control. A significant distortion in the shape of the seminiferous tubule, reduction in the epithelial height, and increased spermatozoa carrying damaged DNA were evident in the animals exposed to sodium arsenite in both treatment durations. It was previously reported that exposure to 60 mg/L of AsO_2_Na in drinking water for 15 days resulted in decreased testicular and epididymal weights, reduced sperm quality, and decreased sperm volume [25]. In another study, exposure to 5 mg/L of sodium arsenite in drinking water for 4 weeks reduced the weight of the testis and epididymal sperm counts and caused considerable degeneration of germ cells [26]. Our findings are in agreement with these studies. Histological examination of the testis revealed seminiferous tubule atrophy, total loss of the spermatogenic layer, absence of spermatozoa in the lumen, and Leydig cell degeneration in animals exposed to sodium arsenite. Meanwhile, the control group displayed active spermatogenesis of all germ cells, such as spermatogonia, and primary and secondary spermatogenesis. Earlier studies found that sodium arsenite treatment caused Leydig cell degeneration, decreased sperm production and spermatid number, and a reduction in the number of epididymal sperm due to oxidative stress [27]. Arsenic activates the hypophysial-adrenocortical axis and enhances the pituitary secretion of adrenocorticotropic hormone. This results in a rise in plasma levels of corticosterone, which suppresses the sensitivity of gonadotrophic cells to the hormone-releasing gonadotropin and thus, prevents the secretion of gonadotropin. High levels of adrenocorticotropic hormone and corticosterone also directly inhibit testosterone production and secretion, by decreasing spermatogenesis and epididymal sperm count [7, 9]. Decreased intratesticular testosterone concentration results in germ cell detachment from the seminiferous epithelium [28]. It may induce germ cell apoptosis because high testosterone levels in the testis are essential for normal spermatogenesis and maintenance of the structure of the seminiferous tubule [29–34].

In this study, sodium arsenite administration in rats demonstrated pathological changes in the liver, including signs of hepatocellular degeneration, inflammation, and pyknosis. The microscopic kidney sections of the rats treated with sodium arsenite revealed tubular degeneration and congestion. Sodium arsenite has been shown to cause histological changes in kidney tissue and increased serum levels of creatinine and urea. The tubular epithelium in kidney sections of rats given sodium arsenite 10 mg/kg BW showed varying degrees of degeneration [35, 36]. Increased concentration of reactive oxygen species [37], decreased efficiency of the antioxidant defense system, and reduced energy levels in cells due to arsenic exposure may result in tissue damage, eventually leading to cell death [38]. It has been documented that sodium-potassium adenosine triphosphate (Na/K ATPase) is responsible for energy-dependent sodium ion extrusion and potassium ion uptake, an essential part of maintaining ionic homeostasis [39]. Oxidative stress induced by arsenic could impair the functioning of the Na/K ATPase pump, resulting in a significant alteration in ion and water transport. This could further lead to the swelling of cells due to fluid accumulation [40]. Decreased Na/K ATPase activity in the plasma membrane of the liver has been observed in mice fed with drinking water containing arsenic [41].

There was significant improvement observed in the sperm count and structure in the animals treated with *M. pruriens* 700 mg/kg BW, and a combination of NAC + *M. pruriens* 530 mg/kg BW in both the treatment groups, respectively. A significant increase in the number of motile sperms in the group treated with 700 mg/kg BW of *M. pruriens* in comparison to M. pruriens administered at other doses was also evident. Long-term exposure in animals to 530 and 700 mg/kg BW of *M. pruriens* demonstrated a better response in reducing the tubule size in comparison to the other treatment doses. Epithelial height of the tubules showed a significant increase in the animals treated with 700 mg/kg BW of *M. pruriens* and a combination of NAC + *M. pruriens* 350 mg/kg BW. In one study, there was a significant increase in the concentration of caudal sperm in rats treated with *M. pruriens* orally at a dosage of 300 mg/kg BW for 14 consecutive days [42]. The positive results observed in the experiment were due to the characteristics of *M. pruriens*.

The seeds of *M. pruriens* are abundant in L-DOPA and metabolites, namely dopamine, epinephrine, and norepinephrine [43]. Dopamine has been suggested to prevent the release of prolactin from the anterior lobe of the pituitary gland [44] and this induces the secretion of gonadotropin-releasing hormone by the hypothalamus and forebrain. This in turn, activates the anterior pituitary gland to secrete gonadotropins resulting in increased testosterone synthesis. The hormone binds to luteinizing hormone receptors present on the cell membrane, inducing activation and the cyclic adenosine monophosphate (cAMP) second messenger system is activated [44]. Increased cAMP levels, due to rapid cholesterol mobilization, are primarily responsible for an increase in steroid production by Leydig cells [45]. Therefore, increased levels of dopamine optimize hormone development, like testosterone, which contributes to improved sexual behavior [46, 47]. Many bioactive constituents, including alkaloids, coumarins, flavonoids, and alkylamines, have been reported to be present in *M. pruriens* [48], which play a significant role in increasing the antioxidant potential in treated males. *Mucun*a *pruriens* has been reported to significantly reduce lipid peroxide levels in infertile men [49, 50] and it is established that lipid peroxidation is a process induced by free radicals and that the lipids in spermatozoa are vulnerable to peroxidation [51, 52]. This antioxidant property may act as a protective effect of *M. pruriens*.

Administration of *M. pruriens* with sodium arsenite demonstrated macrophage activity and improved hepatic structure and sinusoids and inflammatory changes were minimized. Similar results were observed in a study where *M. pruriens* reduced hepatocellular necrosis and prevented cellular infiltration and vacuolation in diabetic rats exposed to 200 mg/kg BW of *M. pruriens* for 28 days, which may be due to its rich antioxidant properties [53]. Alkaloids and saponins are reported to elicit hepatoprotective activity by inhibiting lipid peroxidation [54, 55], thus stabilizing the hepatocellular membrane, preventing cell leakage, and increased hepatic regeneration.

In both treatment groups, graded doses of *M. pruriens* concurrent with sodium arsenite showed substantial damage in the nephron and glomerulus, as observed by histopathological examination. *Mucuna* *pruriens* successfully attenuated the tubular necrosis caused by sodium arsenite in the kidneys of the experimental animals. *Mucuna* *pruriens* proved to be effective due to the potent antioxidant ability of its constituents, which reduced the toxic effects induced by sodium arsenite. This study confirmed that further protection was shown by the simultaneous administration of preventive substances along with arsenic.

## Conclusion

Excessive arsenic ingestion leads to significant damage to various body tissues (liver, kidney, and testes), making the individual prone to further complications. Sodium arsenite-exposed animals showed impaired hepatic and renal function and reproductive toxicity. The supplement of *M. pruriens* (530 and 700 mg/kg BW) with sodium arsenite was found to attenuate the adverse changes observed with arsenic exposure. The study suggests that a diet supplemented with *M. pruriens* can ameliorate the undesirable changes in rats exposed to sodium arsenite.

## Authors’ Contributions

PLC, KLB, and APR: Contributed to the conception and design of the study. PLC: Performed the experiment and wrote the manuscript. PLC, KLB, and APR: Statistical analysis. KLB and APR: Reviewed the manuscript. All authors have read and approved the final manuscript.
